# Primary cervical choriocarcinoma: a case report

**DOI:** 10.3389/fonc.2026.1781668

**Published:** 2026-04-13

**Authors:** Yanlai Xiao, Yiwei Zhang

**Affiliations:** Department of Obstetrics and Gynecology, Hebei Medical University Third Hospital, Shijiazhuang, China

**Keywords:** bevacizumab, cervical, gestational trophoblastic tumor, hCG, primary choriocarcinoma

## Abstract

Primary cervical choriocarcinoma (PCCC) is a sporadic gestational trophoblastic tumor. It originates from the cervical canal, rather than the uterine body. Due to its nonspecific clinical manifestations, it is often easily missed or misdiagnosed. This article presents a case of primary cervical choriocarcinoma. By analyzing its clinical features and treatment process, we aim to improve the diagnosis and treatment of the disease and improve the prognosis of the patient.

## Introduction

Gestational choriocarcinoma (GTN) is a pregnancy-related gynecologic malignancy that originates from placental trophoblast cells, including invasive mole (IM), choriocarcinoma (CC), etc. (1). Among them, choriocarcinoma has the highest degree of malignancy, and the primary site is mostly in the uterus. Primary cervical choriocarcinoma (PCCC) is an extremely rare GTN. Due to its rare and atypical clinical manifestations, it is easily missed or misdiagnosed, resulting in a reduced survival rate (2). Quantitative detection of human chorionic gonadotropin (β-hCG) is an essential diagnostic tool for evaluating abnormal pregnancies. Transvaginal ultrasound (TVUS) is the most commonly used initial imaging modality. In certain cases, Doppler imaging and magnetic resonance imaging (MRI) are employed to further clarify vascular conditions, lesion sizes, and potential myometrial invasion (3, 4). In complex cases, advanced imaging techniques such as positron emission tomography/computed tomography (PET-CT) are utilized for staging assessment and identification of suspected metastatic lesions. For instance, some studies reported (5, 6) the successful identification of metastatic sites in patients with recurrent gestational trophoblastic disease (GTD) using PET-CT, highlighting the significance of multimodal imaging in the diagnosis of GTD. Despite advancements in technology, diagnostic errors remain frequent. This study presents a case of primary cervical choriocarcinoma. By analyzing its clinical features and treatment process, we aim to improve the diagnosis and treatment of this disease and improve the prognosis of patients for clinicians.

## Case report

The 33-year-old female had a history of two full-term deliveries, one medication abortion, and one artificial abortion (in April 2020). She presented with vaginal bleeding on May 7, 2021, and denied abdominal pain or the discharge of vaginal blister-like tissue. Blood hCG levels were measured at 247 mIU/mL (normal <5 mIU/mL), and a gynecological ultrasound indicated “clear endometrium, approximately 0.7 cm thick,” with the bleeding stopping on its own after 10 days. No follow-up blood hCG levels were conducted afterward. Subsequently, there was intermittent vaginal bleeding for three months, which varied in amount, sometimes resembling menstrual flow and other times as bloody discharge. On August 7, 2021, she returned to the community hospital due to vaginal bleeding, and a gynecological ultrasound revealed “clear endometrium, approximately 0.7 cm thick, with a hypoechoic mass near the cervical opening, measuring about 2.8×2.2 cm,” and blood hCG levels of 43,265.6 IU/L. The diagnosis was considered to be “spontaneous abortion? Cervical pregnancy?”, a dilation and curettage (D&C) along with cervical biopsy were performed. During the procedure, a tissue measuring about 3×2 cm was seen on the anterior lip of the cervix, which was red, soft, and highly vascularized. The uterine cavity was approximately 9 cm deep, and a large amount of decidual tissue was aspirated without significant villous tissue observed. Postoperative pathology showed clots and proliferative trophoblastic cells, and immunohistochemical results: cytokeratin AE1/AE3 (+), hCG-β (+), Vimentin (+), ER (–), PR (–), Ki67 (90%), CD10 (+), Desmin (–), CD34 (–), Inhibin-α (+). Combined with the immunohistochemical results, the findings were consistent with choriocarcinoma.

**Figure 1:**
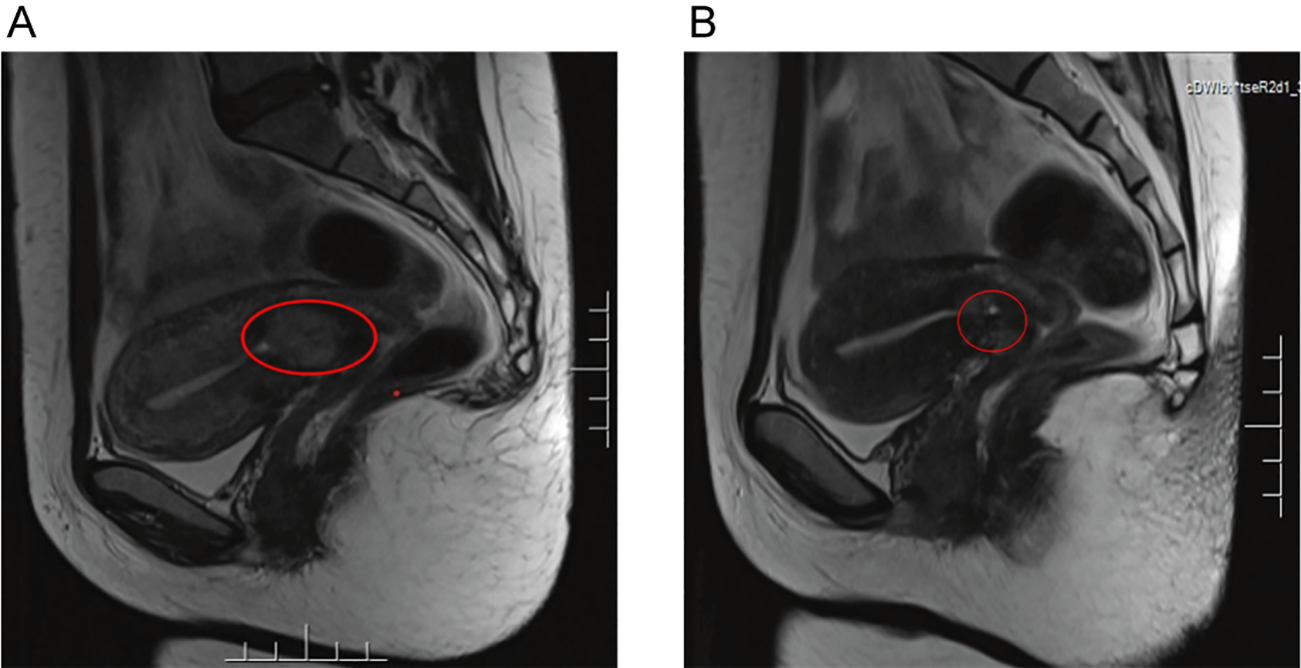
Pelvic MRI shows a lesion centered around the cervix on sagittal T2-weighted Turbo Spin Echo (TSE) image **(A)**. Following chemotherapy, the lesion was significantly reduced in size on sagittal T2- weighted TSE image **(B)**.

**Figure 2:**
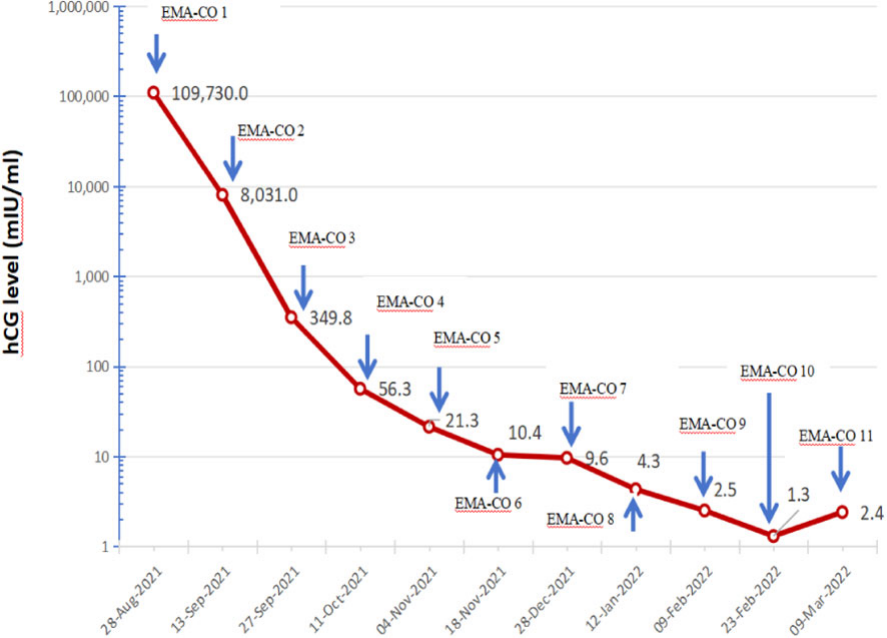
Changes of hCG levels throughout treatment.

Chest CT: scattered solid nodules and metastatic lesions in both lungs. Pelvic MRI revealed a mass in the lower segment of the uterus measuring approximately 3.0 × 2.2 cm (**Figure 1A**). No abnormalities were observed in the upper and lower abdominal CT, enhanced MRI of the brain, abdominal ultrasound, and cervical TCT. According to FIGO/WHO staging and prognosis scoring: 1) Age: 33 years, score 0; 2) Previous pregnancy: miscarriage, score 1; 3) Time since previous pregnancy: more than 12 months, score 4; 4) Pre-treatment blood hCG: 109730mIU/mL, score 4; 5) Maximum tumor size: anechoic area with uneven echogenicity detected within the cervical canal, score 0; 6) Metastatic sites: lungs, score 0; 7) Number of metastatic lesions: none, score 0; 8) Previous failure chemotherapy: none, score 0. Diagnosis: choriocarcinoma (Stage III: 9), indicating a high-risk subtype. The guidelines indicate high-risk GTN is managed with the etoposide, methotrexate, actinomycin D, cyclophosphamide, and vincristine (EMA/CO) chemotherapy regimen. After a total of 11 cycles, herβ-hCG level declined to normal range (**Figure 2**). The patient had no desire for having children, surgical resection was recommended after completion of three rounds of consolidation chemotherapy. However, the patient refused, and she was discharged for follow-up.

**Figure 3:**
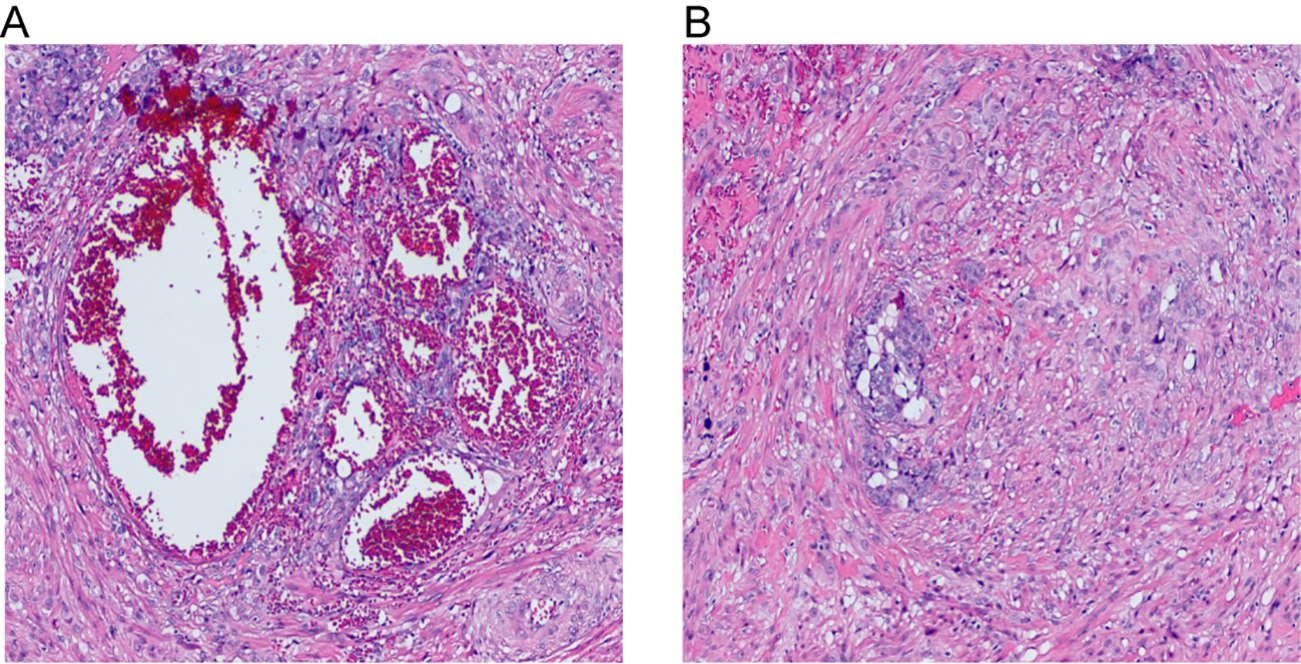
Cytotrophoblast proliferation with cytologic atypia. Areas of hemorrhage with fibrinoid necrosis are noted (**(A)** 10X, **(B)** 10X).

**Figure 4:**
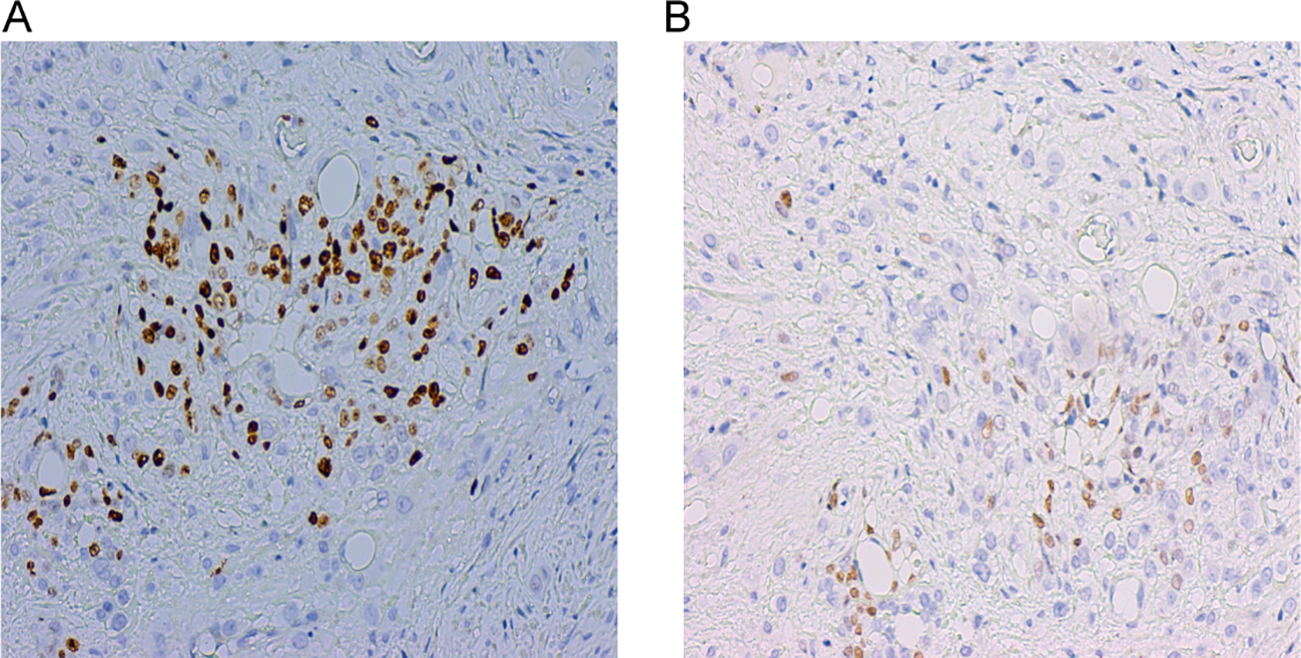
Immunohistochemical studies demonstrate a high Ki-67 index (70%) in tumor cells (**(A)**, 20X). Focal positivity for p63 in tumor cells (**(B)**, 20X).

First recurrence: During follow-up, the serum hCG levels increased to 41.6 mIU/mL four months after chemotherapy discontinuation. Pelvic MRI revealed the mass in the lower segment of the uterus had significantly shrunk compared to before, with a size of 1.4 × 1.0 cm (**Figure 1B**). Diagnosis: Recurrent choriocarcinoma (III:9). The patient had no fertility requirements and underwent laparoscopic total hysterectomy and bilateral salpingectomy. Postoperative pathological examination revealed choriocarcinoma in the cervical canal with associated hemorrhage and necrosis, involving one-third of the cervical canal stroma (**Figures 3A, B**). The parametria and fallopian tubes were ordinary. Endometrium during the proliferative phase. Immunohistochemical results: P57 (+), Ki67 (+70%), CK(Pan) (+), and P63 (focal +) (**Figures 4A, B**). The β-hCG level declined to normal range. The patient received 4 cycles of EMA-CO consolidation chemotherapy, she was discharged for follow-up.

Second recurrence: On May 10, 2023 (after 8 months of chemotherapy cessation), blood hCG levels rose to 28.3 mIU/mL. A gynecological examination revealed a cauliflower-like growth at the left side of the vaginal opening, measuring 0.5 cm in diameter. On May 11, 2023, a biopsy of the lesion was performed, and the postoperative pathology report indicated choriocarcinoma tissue in the lesion. The patient presented with recurrent and metastatic cervical choriocarcinoma, and hCG levels had increased compared to previous measurements. A follow-up PET-CT scan was conducted to assess for metastasis in other organs, which revealed patchy high metabolic lesions on the right side of the vaginal stumps, measuring approximately 1.6×1.4 cm, suggestive of tumor recurrence; no significant abnormality in brain metabolic activity was observed; multiple small nodules were seen in both lungs, with no abnormal metabolic activity noted. Due to multiple recurrences in the patient, bevacizumab treatment was added. Four cycles of the floxuridine, actinomycin D, etoposide and vincristine (FAEV) + bevacizumab chemotherapy regimen were administered, Subsequently, bevacizumab monotherapy was used for maintenance treatment. However, due to persistent hypertension in the patient, with a peak systolic and diastolic pressure of 190/100 mmHg, bevacizumab maintenance therapy was discontinued. The patient has now been off chemotherapy for 3+ years, with no recurrence observed during follow-up to date (**Table 1**).

**Table 1:** Overall treatment process.

Overall treatment process	First treatment	First recurrence	Second recurrence
Date	Aug 28, 2021	Jul 22, 2022	May 10, 2023
hCG (mIU/ml)	109730	41.6	28.3
Chest CT	scattered solid nodules and metastatic lesions in both lungs	--	--
Pelvic MRI	a mass in the lower segment of the uterus measuring approximately 3.0 × 2.2 cm	mass in the lower segment of the uterus had significantly shrunk compared to before, with a size of 1.4 × 1.0 cm	--
PET-CT	--	--	Patchy high metabolic lesions on the right side of the vaginal stumps, measuring approximately 1.6×1.4cm, suggestive of tumor recurrence; multiple small nodules were seen in both lungs, with no abnormal metabolic activity noted. No significant abnormality in brain metabolic activity was observed.
treatment	A dilation and curettage (D&C) along with cervical biopsy + EMA/CO (Eleven cycles)	Laparoscopic total hysterectomy and bilateral salpingectomy + EMA/CO (Four cycles)	a biopsy of the lesion + FAEV + bevacizumab chemotherapy(Four cycles)
Pathological	clots and proliferative trophoblastic cells, and immunohistochemical results: cytokeratin AE1/AE3 (+), HCG-β (+), Vimentin (+), ER (–), PR (–), Ki67 (90%), CD10 (+), Desmin (–), CD34 (–), Inhibin-α (+).	horiocarcinoma in the cervical canal with associated hemorrhage and necrosis, involving one-third of the cervical canal stroma. Immunohistochemical results: P57 (+), Ki67 (+70%), CK(Pan) (+), and P63 (focal +)	pathology report indicated choriocarcinoma tissue in the lesion

## Discussion

Choriocarcinoma is classified into two types based on its origin: gestational choriocarcinoma, which occurs after normal or abnormal pregnancy, and non-gestational choriocarcinoma, which results from the differentiation of trophoblastic cells or somatic cell carcinoma of the reproductive cells. PCCC, which originates from trophoblastic cells within the cervical canal, has an unclear reason for its localization within the cervix rather than the uterine body. It accounts for 1-2% of GTN, with fewer than 200 cases reported in the literature (7). The exact cause remains unclear but may be associated with the malignant transformation of fetal components that form the cervical implant. Additionally, it may be caused by the migration of chorionic cells from a previous pregnancy to this location, followed by a dormant phase during which these cells undergo malignant transformation (4, 8). This may also be related to a history of cervical procedures or inflammation. Moreover, there are reports indicating a connection with chromosomal abnormalities (9).

The patient in this report sought medical attention for irregular vaginal bleeding. Upon examination, cervical polyps and vaginal metastatic nodules were discovered, and a cervical biopsy confirmed choriocarcinoma. However, primary choriocarcinoma of the cervix was not considered at that time. Many literature reviews have shown that PCCC lacks specific clinical manifestations and is easily confused with common cervical diseases (7, 10–12). Gynecological examination reveals an enlarged cervix with a softer texture, and the cervix may exhibit superficial erosion, papillary, or nodular projections that bleed easily upon contact. Some patients may palpate a solid mass in the cervix, and in severe cases, the mass may protrude from the cervical orifice of the uterus. If metastasis occurs, metastatic nodules may be palpable in the vagina or para-uterine tissue. When the nature of the lesions cannot be clearly determined, diagnosis can be confirmed through hCG testing, imaging examinations, and pathological assessments. Serum hCG levels are typically significantly elevated in patients with PCCC. MRI can clearly demonstrate a hypervascular mass within the cervix, characterized by “flow void vessels,” accompanied by multiple areas of hemorrhage and necrosis, while the uterine body remains normal or only slightly displaced (11).

Pathological examination is the gold standard for diagnosing PCCC. It is often confirmed through cervical biopsy; however, biopsy may lead to uncontrollable massive hemorrhage, and it is recommended to be performed under conditions where emergency hemostasis (such as interventional embolization) is available. The pathological features of PCCC are consistent with those of classic choriocarcinoma, characterized by the abnormal proliferation of cytotrophoblasts, syncytiotrophoblasts, and intermediate trophoblasts without the formation of villous structures, accompanied by extensive hemorrhage and necrosis (13). Immunohistochemistry showed strong positivity for β-hCG, GATA3, P63, and inhibin-α, and a high Ki-67 proliferation index (typically >50%). For diseases that are difficult to differentiate, monitoring serum β-hCG levels and reviewing immunohistochemical pathology slides can aid in an accurate diagnosis (14). Finally, differentiating between gestational and non-gestational choriocarcinoma is crucial, as the non-gestational form responds poorly to chemotherapy (15). This is achieved by STR genotyping and finding unique paternal genetic material in the tumor (16). We did not have the technical means for this analysis, therefore we declared this PCCC as being of gestational origin based on data from an miscarriage 15 months previously.

The therapeutic guidelines for PCCC follow the general strategy of Gynecological Oncology, considering the unique anatomical features of the cervix. A comprehensive treatment strategy was implemented, primarily centered on chemotherapy, and integrated with surgical intervention, radiotherapy, and targeted therapy, thereby significantly improving therapeutic outcomes. The choice of chemotherapy regimen can be determined using the FIGO scoring system. The EMA-CO regimen is the current standard for treating high-risk GTN, achieving a response rate of 80% to 90% (17, 18). Some patients undergo hysterectomy due to vaginal bleeding, recurrence, or chemoresistant lesions. These findings emphasize the importance of surgery in the diagnosis and treatment methods of PCCC (11). This case underwent a total hysterectomy due to recurrence, and the postoperative pathological diagnosis confirmed PCCC. Thus, oncological safety should be considered when preserving fertility, surgical treatment for PCCC should not be neglected.

The overall complete response rate of PCCC was 95%, with a recurrence rate of 13%, which was higher than that of conventional GTN (6.5%). Over the last decade, the landscape of solid tumor treatment has been transformed by the inhibition of immune checkpoints, achieving satisfactory therapeutic outcomes (19). In gestational trophoblastic tumors (GTNs), the expression level of programmed death-ligand 1 (PD-L1) is quite high (20, 21). Early use of pembrolizumab and avelumab had shown encouraging results, including complete and durable remission in multidrug-resistant diseases (22–24). However, due to economic considerations, bevacizumab was added for targeted consolidation therapy for this case. The patient later discontinued bevacizumab maintenance therapy due to severe hypertension, with blood pressure returning to normal after cessation. The patient has been off chemotherapy for 3+ years and remains in complete remission upon follow-up, which is consistent with the literature reports (25).

## Conclusion

Due to its clinical rarity and non-specific symptoms, early diagnosis of cervical choriocarcinoma is difficult. In the differential diagnosis of cervical lesions in women of reproductive age, β-hCG measurement, pelvic ultrasound, MRI, and cervical biopsy with histopathological examination may be helpful. Histopathological diagnosis is particularly important for cervical choriocarcinoma. Chemotherapy is the preferred treatment. For patients with recurrence, surgery combined with targeted chemotherapy can improve the prognosis of patients with drug-resistant recurrence. Future research needs to accumulate more cases to clarify the etiology and pathogenesis, explore more optimized treatment protocols, and simultaneously enhance the clinical awareness of medical professionals regarding this disease to improve the diagnosis and cure rates of patients.

## Data Availability

The datasets presented in this study can be found in online repositories. The names of the repository/repositories and accession number(s) can be found below: The original contributions presented in the study are included in the article/supplementary material. Further inquiries can be directed to the corresponding author.
